# Geschlossene Heime für Menschen mit geistiger oder seelischer Behinderung – Charakteristika von Bewohnenden anhand von Aktenanalysen

**DOI:** 10.1007/s00115-022-01437-5

**Published:** 2023-01-24

**Authors:** Peter Brieger, Tanja Henking, Thomas Schmitt-Schäfer, Malte Klemmt, Ernst Daniel Röhrig, Johannes Hamann

**Affiliations:** 1 kbo-Isar-Amper-Klinikum Region München, Akademisches Lehrkrankenhaus der LMU, Vockestr. 72, 85540 Haar, Deutschland; 2 Hochschule für angewandte Wissenschaften Würzburg-Schweinfurt, Würzburg, Deutschland; 3 transfer – Unternehmen für soziale Innovation, Wittlich, Deutschland; 4 Klinik für Psychiatrie und Psychotherapie, TU München, München, Deutschland; 5 Bezirksklinikum, Ärztliche Direktion, Mainkofen, Deutschland

## Hintergrund

Geschlossene Heime für Menschen mit seelischen und geistigen Behinderungen sind in der Bundesrepublik Deutschland als Einrichtungen der stationären Eingliederungshilfe in der Versorgung von Menschen mit seelischer oder geistiger Behinderung eine kritisch diskutierte Realität. Es fehlen empirische Erkenntnisse zu den Charakteristika der Bewohnenden wie auch zu Aspekten der Prozess- und Ergebnisqualität.

Das Bayerische Staatsministerium für Gesundheit und Pflege vergab TH, TSS und PB den Auftrag zur Erstellung eines Gutachtens zu freiheitsentziehenden Maßnahmen in Einrichtungen für (erwachsene) Menschen mit Behinderung in Bayern (www.fem-bayern.de). Im Rahmen dieser Untersuchung erfolgte eine Analyse der Dokumente von Bewohnenden mit besonderem Fokus auf Menschen mit geistiger oder seelischer Behinderung in geschlossenen Einrichtungen.

Anhand der dort erhobenen Daten wurden im Rahmen einer Sekundäranalyse folgende Fragen beantwortet:

1. Welche Charakteristika zu den Bewohnenden geschlossener Wohnheime sind aus der Studie ersichtlich?
2. Waren andere Auffälligkeiten ersichtlich, die Rückschlüsse auf die Verfahrensweisen unter Berücksichtigung der Menschenrechte in geschlossenen Einrichtungen ergeben?

## Methodik

Gemäß des Studienprotokolls [6] wurden nach einer onlinegestützten Gesamterhebung aller Heime für Menschen mit Behinderung in Bayern (*n* = 850) 5 geschlossene bzw. teilgeschlossenen Wohngruppen in Heimen exemplarisch für die Durchführung von teilnehmenden Beobachtungen, Dokumentenanalysen und einrichtungsbezogenen Fokusgruppen benannt (weitere Publikationen werden folgen). Diese waren von den Studienleitungen hinsichtlich regionaler Verteilung, Träger und innerer Charakteristika aus einer Gruppe ausgewählt worden, die ihre Teilnahme angeboten hatten. PB und JH untersuchten die Einrichtungen 2021/22 als Fachärzte für Psychiatrie und Psychotherapie mittels einer Begehung. Im Rahmen der Dokumentenanalyse wurden Unterlagen hinsichtlich Unterbringung, Kostenübernahme, medizinische Unterlagen und die Dokumentation des Wohnverlaufs erfasst. Es wurden Gespräche mit den therapeutischen Leitungen geführt sowie die jeweils zu untersuchende geschlossene Station begangen. Eine direkte Untersuchung der Bewohnenden erfolgte nicht. Die Erfassung der Daten erfolgte mithilfe eines standardisierten Erhebungsprotokolls. Hinsichtlich der Medikation wurde diese nach Kategorien gemäß der Roten Liste klassifiziert (www.rote-liste.de). Es wurde bewertet, inwieweit die eingesetzte Medikation und ihre Dosierung gemäß der Hauptdiagnose der jeweils geltenden S3-Leitlinie AWMF folgt (ja – nein – Grenzfall). Die Einwilligung der Betroffenen bzw. ihrer rechtlichen Betreuer bzw. Vorsorgebevollmächtigten wie auch ein Votum der Ethikkommission der LMU München wurden eingeholt. Die Dokumentationen wurden durch die beiden Untersucher wechselseitig intervidiert.

## Ergebnisse

Für 46 Personen konnten die Akten untersucht werden (zwischen 7 und 12 Akten pro Einrichtung). Entsprechende Charakteristika sind in Tab. 1 und 2 dargestellt.

**Table 1:** 

Variable	Mittelwert	Minimum/Maximum	Bemerkung
Alter	40,1	18–82	–
Aufenthaltsdauer im Heim (Jahre)	11,5	0–57	6 Personen länger als 30 Jahre; Männer 14,6 Jahre, Frauen 7,9 Jahre. Menschen mit einer geistigen Behinderung 15,4 Jahre vs. 3,6 Jahre (seelische Behinderung)

**Table 2:** 

Variable	Verteilung	Weitere Beobachtungen
Geschlecht (m/w)	25/21	Sehr unterschiedlich: in einer Einrichtung 8 von 12 Frauen, in einer anderen 7 von 8 Männer
Herkunft (derselbe bayerische Bezirk/anderer Bezirk/anderes Bundesland)	12/16/9	Menschen mit geistiger Behinderung stammten häufiger aus anderen Bezirken oder Bundesländern (61,3 %) als Menschen mit seelischer Behinderung (40,0 %)
Diagnosen (ICD-10 Kategorien – nicht exklusiv)	F70 (Intelligenzminderung): 31 F2 (psychotische Erkrankung): 19 F1 (Sucht): 8 F84 (Autismusspektrum): 8 F60.31 (Borderline-Persönlichkeitsstörung): 6	–
Psychopharmakadauermedikation (ja/nein)	46/2	Neuroleptika bei 38 Personen

*ICD* International Classification of Diseases, *m* männlich, *w* weiblich

Bei 7 der mit Psychopharmaka behandelten Bewohnenden lag die Indikation des Psychopharmakons außerhalb der gültigen Leitlinie (z. B. Dauermedikation mit Benzodiazepinen oder Einsatz von Neuroleptika bei Borderline-Persönlichkeitsstörung), bei 9 Personen wurde der Einsatz der Medikation als Grenzfall eingeschätzt (v. a. im Bereich von Menschen mit geistiger Behinderung). Bei 28 Personen entsprach der Einsatz den Leitlinien.

Insgesamt 42 der 46 Bewohnenden waren zivilrechtlich (nach § 1906 Abs. 1 BGB) im Heim untergebracht, bei 2 lag eine „freiwillige“ Zustimmung vor (bei einem durch den Betreuer allerdings ohne richterliche Genehmigung) und bei 2 bestand keine Weglaufgefahr aufgrund körperlicher Immobilität, sodass eine Unterbringung nicht erforderlich wurde. Gerichtsbeschlüsse waren in der Mehrzahl formelhaft (Textbausteine). In der Mehrzahl waren die Gutachten und Gerichtsbeschlüsse alle 2 Jahre nahezu identisch.

## Zusammenfassung und Beurteilung

Bei den Unterbringungsverfügungen der Gerichte war erkenntlich, dass diese häufig festen Textbausteine folgten – basierend auf Gutachten identischer Gutachter mit nahezu gleichen Gutachtenstexten, die alle 2 Jahre perpetuiert wurden – manches Mal 10 Jahre und länger. Problematisch erschien in Gerichtsbeschlüssen die Nutzung des „Time-out-Raumes“. Von manchen Gerichten wurde eine Unterbringung im Time-out-Raum als freiheitsentziehende Maßnahme genehmigt. Dabei wird unseres Erachtens ein Time-out Raum als primär therapeutisches Angebot als Isolationszimmer missverstanden [2].

Bezüglich der Medikation waren einzelne Medikationen auffällig, da insbesondere im Bereich der Menschen mit geistiger Behinderung Zulassungen und Leitlinienempfehlungen begrenzt sind.

Fast jeder fünfte Bewohnende stammte aus einem anderen Bundesland, sodass die Einbindung in die ursprünglichen sozialen Beziehungen, Familien und das Normalisierungsgebot nicht unterstützt werden. Manche Bewohner zogen vor mehr als 50 Jahren aus anderen Bundesländern ins geschlossene Heim nach Bayern und leben da heute noch.

Dass die Anwendung freiheitsentziehender Maßnahmen ein basales Problem von Pflegeheimen ist, wurde wiederholt dargestellt [3]. Heime für Menschen mit seelischen und geistigen Beeinträchtigungen fanden diesbezüglich weniger Beachtung [1]. Die PINO-Studie [7] hat Informationen zur Situation von Menschen mit geistiger Behinderung in Heimen veröffentlich – inklusive der Häufigkeit von FEM, während die ZIPHER-Studie [5, 8] bundesweit Struktur- und Prozessdaten stationärer Wohneinrichtungen berichtete. Unsere Daten ergänzen diese Ergebnisse mit anderem Fokus.

Die methodischen Grenzen dieser Untersuchung sind offensichtlich: Wir haben in nur einem Bundesland in 5 Heimen 46 Personen anhand ihrer Akten untersucht, was ein sehr begrenztes Spektrum eines sehr viel größeren Bereiches widerspiegelt. Wir gehen davon aus, dass Heime an der Untersuchung teilgenommen haben, die eine kritische Haltung zu freiheitsentziehenden Maßnahmen haben und an Verbesserungsprozessen interessiert sind.

In Kliniken hören wir wiederholt die Überzeugung, es gäbe zu wenig „geschlossene“ Heimplätze, und wir wünschen uns mehr Bestreben, hier Alternativen zu gestalten. Goffmans Kritik der „totalen Institution“ [4] verpflichtet zu mehr politischer und Fachaufmerksamkeit sowie repräsentativen Studien und Änderungsansätzen. Dabei stellte sich auch die Frage nach der Perspektive und übergreifenden Hilfeplanung, wenn z. B. eine 22-jährige junge Frau mit einer Borderline-Persönlichkeitsstörung mehrere Jahre in einem geschlossenen Heim lebte und zum dritten Mal mit dem nahezu „identischen Gutachten“ für weitere 2 Jahre untergebracht wird.

Nach der 2008 in Kraft getretenen UN-Behindertenrechtskonvention soll gewährleistet sein, dass Menschen mit Behinderungen gleichberechtigt die Möglichkeit haben, „ihren Aufenthaltsort zu wählen und zu entscheiden, wo und mit wem sie leben, und nicht verpflichtet sind, in besonderen Wohnformen zu leben“ (Artikel 19). Geschlossene Heime sind möglicherweise mangels Alternative unumgängliche, aber in diesem Sinne problematische Orte, da sie offenkundig im Konflikt mit Selbstbestimmung, Teilhabe und Normalisierung stehen.

## Fazit für die Praxis

- Es fehlt an Erkenntnissen zu den Bewohnenden (teilweise) geschlossener Einrichtungen wie auch an politischer und Fachaufmerksamkeit.
- Gerade Ärzte und Ärztinnen in Kliniken sollten die Realität und insbesondere die Grenzen „geschlossener Heime“ kennen.
- In Gutachten und gerichtlichen Unterbringungsbeschlüssen für die Unterbringung in geschlossenen Heimen fehlte in dieser Untersuchung in der Mehrzahl eine Auseinandersetzung mit der Notwendigkeit der und Alternativen zur Unterbringung.
- Kliniken sollten ihren Fokus auf die Suche nach alternativen Versorgungskonzepten legen, um Unterbringungen in „geschlossene Heime“ zu vermeiden. Wohnortferne Unterbringungen sollten in der Regel vermieden werden.
